# Rab32 connects ER stress to mitochondrial defects in multiple sclerosis

**DOI:** 10.1186/s12974-016-0788-z

**Published:** 2017-01-23

**Authors:** Yohannes Haile, Xiaodan Deng, Carolina Ortiz-Sandoval, Nasser Tahbaz, Aleksandra Janowicz, Jian-Qiang Lu, Bradley J. Kerr, Nicholas J. Gutowski, Janet E. Holley, Paul Eggleton, Fabrizio Giuliani, Thomas Simmen

**Affiliations:** 1 Department of Cell Biology, University of Alberta, Edmonton, Canada; 2 Department of Medicine, Division of Neurology, University of Alberta, Edmonton, Canada; 3 Department of Laboratory Medicine and Pathology, University of Alberta, Edmonton, Canada; 4 Department of Anesthesiology and Pain Medicine, University of Alberta, Edmonton, Canada; 5 University of Exeter Medical School & Neurology Department, Royal Devon & Exeter Hospital, Exeter, UK; 6 Present address: Alberta Diabetes Institute, University of Alberta, Edmonton, Canada

**Keywords:** Multiple sclerosis, Endoplasmic reticulum, Mitochondria, Unfolded protein response (UPR)

## Abstract

**Background:**

Endoplasmic reticulum (ER) stress is a hallmark of neurodegenerative diseases such as multiple sclerosis (MS). However, this physiological mechanism has multiple manifestations that range from impaired clearance of unfolded proteins to altered mitochondrial dynamics and apoptosis. While connections between the triggering of the unfolded protein response (UPR) and downstream mitochondrial dysfunction are poorly understood, the membranous contacts between the ER and mitochondria, called the mitochondria-associated membrane (MAM), could provide a functional link between these two mechanisms. Therefore, we investigated whether the guanosine triphosphatase (GTPase) Rab32, a known regulator of the MAM, mitochondrial dynamics, and apoptosis, could be associated with ER stress as well as mitochondrial dysfunction.

**Methods:**

We assessed Rab32 expression in MS patient and experimental autoimmune encephalomyelitis (EAE) tissue, via observation of mitochondria in primary neurons and via monitoring of survival of neuronal cells upon increased Rab32 expression.

**Results:**

We found that the induction of Rab32 and other MAM proteins correlates with ER stress proteins in MS brain, as well as in EAE, and occurs in multiple central nervous system (CNS) cell types. We identify Rab32, known to increase in response to acute brain inflammation, as a novel unfolded protein response (UPR) target. High Rab32 expression shortens neurite length, alters mitochondria morphology, and accelerates apoptosis/necroptosis of human primary neurons and cell lines.

**Conclusions:**

ER stress is strongly associated with Rab32 upregulation in the progression of MS, leading to mitochondrial dysfunction and neuronal death.

**Electronic supplementary material:**

The online version of this article (doi:10.1186/s12974-016-0788-z) contains supplementary material, which is available to authorized users.

## Background

At an advanced stage of MS, immunomodulating therapies are no longer effective, highlighting the need to understand the molecular basis of this disease. Like other neurodegenerative diseases that are associated with mitochondrial impairment [1], MS mitochondria can be dysfunctional, especially during disease progression [1] and its neurodegenerative phase [2, 3]. For instance, mitochondria no longer respire normally in progressive MS patients [4]. Dysfunctional mitochondria produce reactive oxygen species (ROS). As a consequence, mitochondrial ROS promote inflammation and shift mitochondrial dynamics towards fission [5]. This latter process requires dynamin-related protein 1 (Drp1), a ubiquitous guanosine triphosphatase (GTPase) [6]. While Drp1 is essential for post-mitotic neurons [7], its excessive activity can result in apoptosis [8]. Indeed, mitochondria increase in number in MS neurons undergoing demyelination [9], thus accelerating axonal degeneration [10]. Upstream causes of these mitochondrial defects are largely unknown. One potential mechanism involves the uncontrolled release of Ca^2+^ ions from the main cellular Ca^2+^ store, the endoplasmic reticulum (ER), within the diseased neurons. This intracellular signaling mechanism ultimately promotes cell death via a mitochondrial mechanism [11, 12]. Lethal ER-mitochondria Ca^2+^ transfer is observed for instance following axotomy [13] or spinal cord injury [14].

In addition to disrupted mitochondrial dynamics, the induction of ER stress is another hallmark of MS [15, 16]. This process not only promotes ER-mitochondria crosstalk in general by increasing the apposition of the ER and mitochondria at the so-called mitochondria-associated membrane (MAM) [17–19], but could also promote neuronal death, thus contributing to the MS pathology [20].

A prominent MAM regulatory protein is Rab32. This GTPase localizes to the ER and mitochondria [21, 22], where it regulates ER-mitochondria interactions and mitochondrial dynamics [23]. Rab32 is induced upon brain inflammation in a mouse model [24]. Consistent with an important role in neuroinflammation, our data indicate that ER stress induces Rab32 and occurs in the MS brain. These findings increase our understanding of Rab32 role in impairment of neuronal mitochondrial dynamics and cell survival.

## Methods

### Antibodies

Antibodies used in this study were purchased as follows: anti-actin, anti-phospho-Drp1 Ser637 (Cell Signaling, Danvers, MA), anti-amyloid precursor protein, anti-glucose-regulated protein of 94 kDA (GRP94), anti-α tubulin, anti-receptor-interacting protein kinase (RIPK) (EMD-Millipore, Billerica, MA), anti-immunoglobulin-binding protein/glucose-regulated protein of 78 kDA (BiP/GRP78) (BD Biosciences, Franklin Lakes, NJ), anti-BiP/GRP78, anti-Drp1 (abcam, Cambridge, UK), anti-CD68 (Dako/Agilent, Markham, ON), anti-CCAAT/enhancer-binding protein (C/EBP) homologous protein (CHOP), anti-GRP75 (Pierce/Thermo, Waltham, MA), anti-CHOP (Enzo, Farmingdale, NY), anti-phosphofurin acidic cluster sorting protein 2 (PACS-2) (Protein Tech, Chicago, IL), anti-Rab32 (Sigma/Aldrich, St. Louis, MO), and anti-FLAG (Rockland, Limerick, PA). The antibody against calnexin has been described previously [25].

### Isolation and maintenance of primary neuronal cultures

Cultures of human fetal neurons (HFN) were generated from 15–19-week fetal brains (obtained with consent from the University of Alberta Ethics Committee) as described [26].

### Human frozen brain tissues and EAE mice tissue

For immunohistochemistry, snap-frozen blocks of post-mortem normal control (NC) or MS cerebral sub-ventricular deep white matter samples were obtained from the NeuroResource Tissue Bank, UCL Institute of Neurology, London, and UK MS Biobank, with next-of-kin informed consent for tissue donation and ethical approval from Central London REC1 (I.D.08/H0718/62) and approval for the study from the Local Research Ethics Committee (I.D.04/Q2102/111), UK MS brain bank charity number 1139257. A total of 12 MS patients (9 females, 3 males) who had been affected from secondary progressive (10), primary progressive (1) or relapsing progressive MS were used for this part of the study. Control tissue from individuals who had not been affected by disease (7) and 2 individuals who had been affected by Parkinson’s disease was also examined. Control patients died of non-inflammatory diseases (cardiac failure, lung cancer, bladder cancer, prostate cancer, tongue cancer, myelodysplastic syndrome; for two control cases, the cause of death was not known). Further information is contained in Additional file 1.

For Western blot and immunofluorescence analysis, tissues of two frozen MS brains (patient 1: secondary progressive MS, aged 54, male; patient 2: relapsing-remitting MS, aged 45, male) were obtained from the MS Tissue Bank at the University of Alberta. Post-mortem brain tissues were collected and processed as described [27]. Frozen brain and spinal cord tissues of triplicate experimental autoimmune encephalomyelitis (EAE) mice, an animal model of MS, were generated with proper approvals as described [28]. Control samples showed no signs of nervous disease. Disease peak samples were from clinical grade 1, whereas post-peak samples were from clinical grade 4 (hind limb paralysis at time of dissection).

### Lysate preparation and analysis from tissues and cell lines

Tissue lysates were prepared from the human frozen brain as well as from the spinal cords of EAE mice in 1× sodium dodecyl sulphate (SDS) extraction buffer (0.125 M Tris-HCK pH 6.8, 2% SDS, 10% glycerol, 5% β-ME), followed by sonication on a 550 Sonic Dismembrator (Fisher Scientific, Ottawa, ON). Supernatants were collected, and protein concentrations were measured by NanoDrop Spectrophotometer ND1000 (Thermo/Life Technologies) at an absorbance of 280 nm. Cellular lysates from SH-SY5Y cells were prepared as described [25].

### RT-PCR

SH-SY5Y cells were cultured in mild hypoxia (4% O_2_, grown in the presence of 4% O_2_, 5% CO_2_ balanced N_2_, as is typical for brain tissue) in the presence of thapsigargin. After 24 h in culture, total RNA was extracted. The primers used for RT-PCR were as follows: Rab32 forward AGCAGGACTCTGGTGCGCCTG (position 211-231); Rab32 reverse CGGGCAGCTTCCTCTATGTTTATGTTATC (position 557-529). The result was normalized against the ribosomal 18S.

### Immunohistochemistry

Sample sections were stained with hematoxylin and eosin (H&E) and luxol fast blue (LFB) as described [27]. Lesions were classified into acute (referring to tissue phenotype, see below), sub-acute, and chronic on the basis of the number and distribution of oil red-O-positive macrophages, the extent of demyelination, cellularity in the borders and parenchyma of lesions, and perivascular cuffing as described in our previous work [29]. Briefly, acute lesions were identified via demyelination, invading macrophages, hypercellularity at the lesion border, and cuffing around the blood vessels. Sub-acute lesions showed a demyelinated plaque with fewer macrophages, mostly at the lesion border, and less perivascular cuffing. A chronic lesion consisted of a hypocellular demyelinated plaque completely lacking of oil red-O-stained macrophages. Examination of MS brain tissue for Rab32 expression in specific cell types was performed employing enzyme immunohistochemistry using a Vectastain ABC system® (Vector Laboratories, Peterborough, UK), as described [30].

### Transfection of constructs and shRNA, immunofluorescence, and quantification of apoptosis

mCherry-labeled Rab32 shRNA psi-mH1 plasmids (HSH001118) as well as scrambled control (CSHCTR001) were purchased from Genecopoeia (Rockland MD). FLAG-tagged Rab32 constructs were expressed from pcDNA3 as published [22] (wt, wild type; Q85L, dominant-active; T39N, dominant-negative) or transferred into the bi-cistronic pIRES2-EGFP plasmid (Clontech-Takara, Mountain View, CA) that allows for the expression of any protein, in parallel with nuclear EGFP. To do so, the described constructs contained in pcDNA3 were PCR-amplified using the SP6 and TS484 (ATATGCTAGCACCATGGACTACAAGGACGACGATGACAAG) oligos following cuts with the 5’ Nhe1 and 3’ Xho1 sites. Primary neurons or SH-SY5Y neuronal cell lines were transfected by nucleofection (Lonza, Mississauga, ON). Immunofluorescence was performed as described [25]. To assay neurotoxicity, nuclear EGFP was used to identify transfected HFNs and SH-SY5Y. Apoptosis was then detected by Cy5-annexin V binding (BD Biosciences). Assays were repeated in the presence of bafilomycin (100 nM, Sigma-Aldrich), necrostatin-1 (Nec-1, 50 μM, Cayman Chemical), carbobenzoxy-valyl-alanyl-aspartyl-[O-methyl]- fluoromethylketone (zVAD-fmk) (10 μM, Enzo Life Sciences, Farmingdale, NY), or with a combination of nec-1 and zVAD-fmk.

### Immunogold labeling

Cells were rinsed in PBS and fixed in 3% paraformaldehyde and 0.05% glutaraldehyde (GA) containing 2% sucrose. Next, free aldehyde groups were quenched with ammonium chloride (50 mM), and samples were permeabilized with saponin (0.1%). The samples were blocked (PBS + 1% BSA + 0.05% FSG + Saponin 0.05%) for an hour and then were incubated with mouse anti-FLAG in the blocking buffer overnight in a wet chamber. Following washes (0.2%BSA + 0.05%FSG + 0.05% saponin), the samples were incubated with the secondary antibody (Fluoronanogold Anti-mouse Fab’Alexa Fluor 488, cat. 7202; Nanoprobes, NY) for 3 h at RT and washed with PBS three times. The samples then were fixed (2% GA in PBS + 2% sucrose) for an hour, followed by three rinses in water. Following a 1-min incubation with GoldEnhance EM Plus (Cat. 2114; Nanoprobes, NY), the samples were rinsed in water, scraped in 100 mM sodium cacodylate and pelleted. The pellet was incubated for 1 h with osmium tetroxide (1%), followed by overnight staining with uranyl acetate. After dehydration in increasing concentrations of ethanol and then propylene oxide treatments, the pellets were transferred to resin (Embed 812 kit, cat. 14120; Electron Microscopy Sciences, Hatfield, PA) and incubated at 60 °C for 48 h. Blocks were sectioned (70 nm) using Ultracut E (Reichert Jung) and imaged with a Philips 310 electron microscope, equipped with a digital camera (Mega View III Soft Imaging System, Emsis Gmbh, Muenster, Germany).

## Results

### Rab32 parallels ER stress within MS patient and EAE brains

**Fig. 1:**
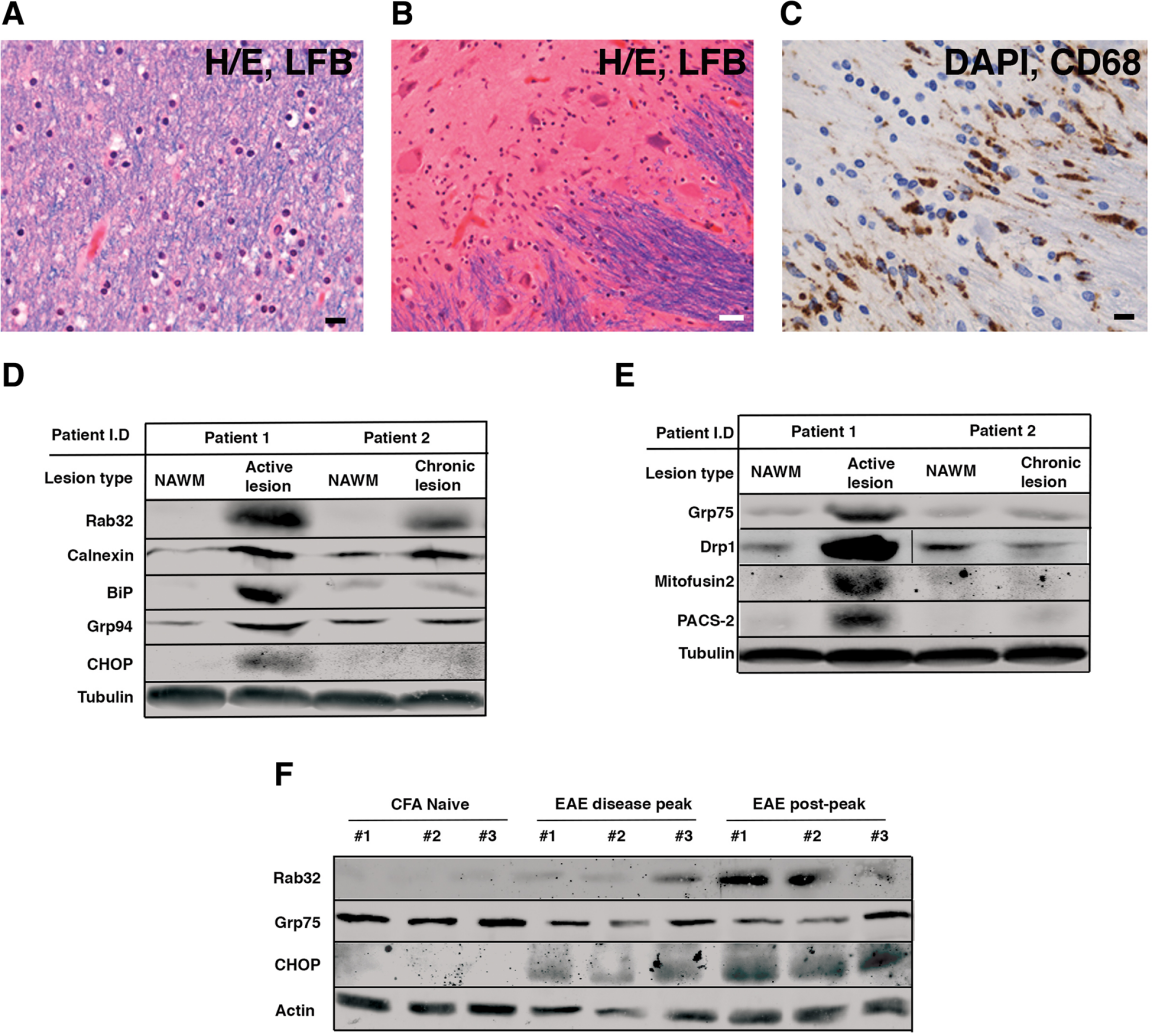
Proteomic characterization of MS brain tissues. **a** Normal-appearing white matter (NAWM) in the right frontal lobe exhibiting intact LFB staining for myelin. **b** Identification of lesion and NAWM areas in autopsy tissue sections of an MS patient brain derived from a 44-year-old male patient. Hematoxylin and eosin staining in a chronic lesion in the pons shows a hypocellular center (*upper left*) containing neuronal cells with diminished LFB staining for myelin and hypercellular edge. **c** Abundant CD68-immunoreactive macrophages/microglia are detected in the hypercellular edge of the active lesion. **d** Western blotting analysis showing the amounts of Rab32, together with ER stress-related markers (calnexin, BiP/GRP78, GRP94, and CHOP (patient 1: secondary-progressive MS, patient 2: relapsing-remitting MS)). **e** Western blotting analysis showing the amounts of MAM-related proteins GRP75, Drp1, mitofusin2, and PACS-2. **f** Expression of Rab32 associated with selected marker proteins in EAE (GRP75 and CHOP) in tissues obtained from the spinal cords of EAE mice. Triplicate samples from CFA naive, EAE disease peak (clinical grade 1), and EAE post-peak (clinical grade 4) are shown

Rab32 is enriched on the ER and mitochondria [31], where it determines various aspects of ER-mitochondria crosstalk [21, 23]. The recent discovery that Rab32 expression increases during brain inflammation in mice [24] and the connection between ER-mitochondria crosstalk and inflammation [32, 33] led us to hypothesize that Rab32 might play a role in the MS pathology. Thus, we examined autopsy tissue sections from the MS patient brains (Fig. 1a–c) for Rab32 expression. These results demonstrated that Rab32 was increased in lesions of MS brain tissues (Fig. 1d). Importantly, Rab32 was higher in active lesions where infiltrating macrophages and resident microglia were present. Consistent with the reported low expression of Rab32 in brain tissue [34, 35], very low levels of Rab32 were noticed in the normal-appearing white matter (NAWM, Fig. 1d). We next tested whether this increase in Rab32 paralleled an increase of proteins functionally connected to Rab32, including ER chaperones and proteins regulating ER-mitochondria interactions.

Consistent with the previous identification of ER stress as a hallmark of the MS CNS [16, 36], we detected increased expression of calnexin, BiP/GRP78, GRP94, and the CCAAT/enhancer-binding protein (C/EBP) homologous protein (CHOP) in active, but not chronic lesions (Fig. 1d). Next, we tested whether other MAM regulatory proteins were upregulated as well. We probed for the tether proteins GRP75 and Mfn2 the MAM-associated mitochondria fission GTPase Drp1 and the MAM enrichment factor phosphofurin acidic cluster sorting protein 2 (PACS-2). Western blotting showed that all of the above proteins showed increased expression in active, but not chronic MS brain lesions (Fig. 1e). We extended our investigation into the animal model of MS, experimental autoimmune encephalomyelitis (EAE). Western blotting showed that high levels of brain-localized Rab32 occurred in the peak and post-peak period of EAE (Fig. 1f), reflecting the induction of Rab32 in both active and chronic lesions of MS brain.

### Cell-type specific localization of Rab32 expression

**Fig. 2:**
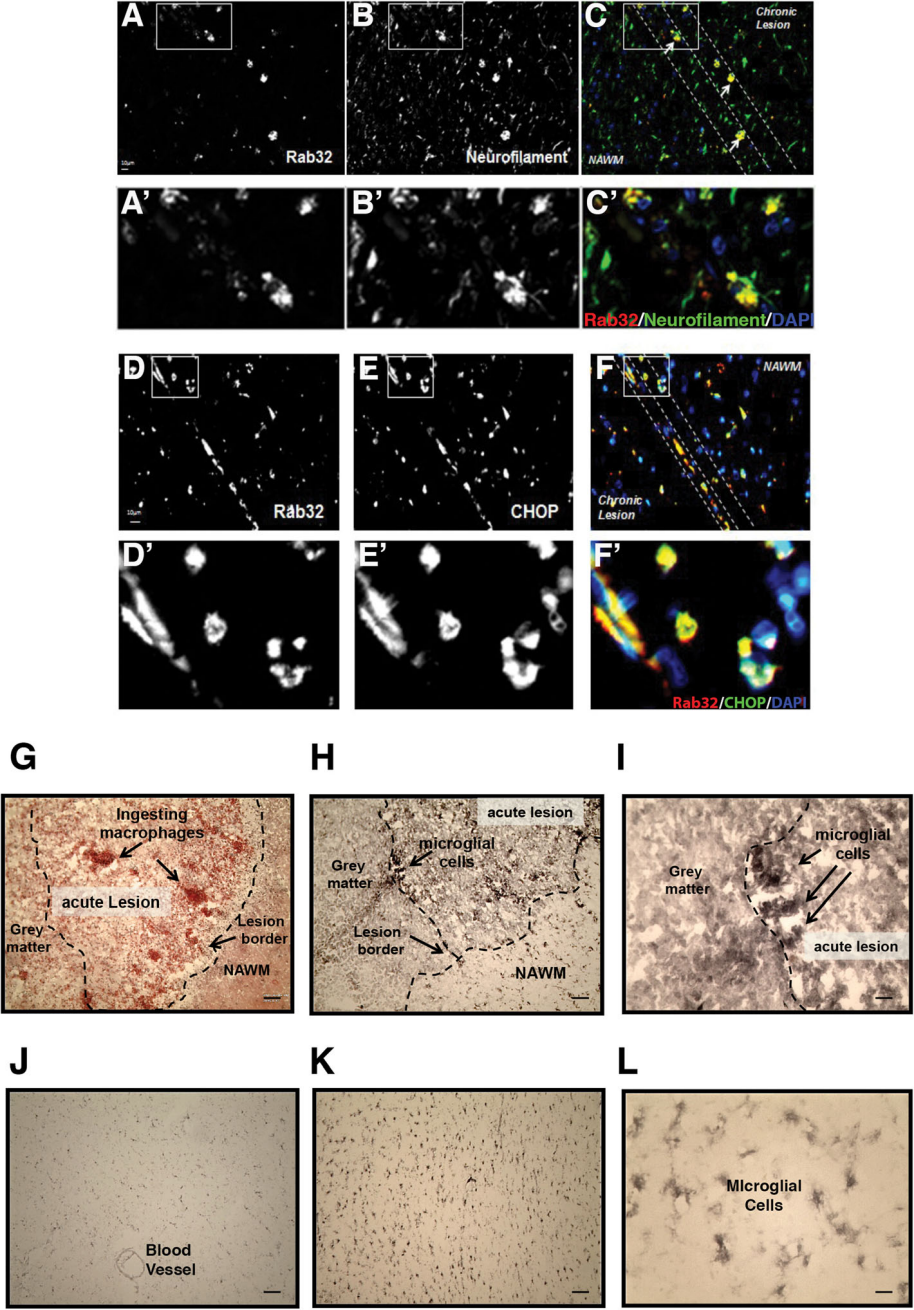
Rab32 localization in active MS lesions. **a**–**f** Immunofluorescence stainings of patient brain tissue from secondary progressive MS showing Rab32 (**a**, **d**), neurofilament (**b**), merged Rab32/neurofilament, including DAPI (**c**), CHOP (**e**) and merged Rab32/CHOP, including DAPI (**f**). Enlarged areas in **a**–**f** are shown below (**A’**–**F’**). Active chronic lesion, lesion border, and NAWM were identified using H&E and LFB stain of adjacent sections as described in Fig. 1**a**–**c**. **g**–**l** Representative images from a 12-patient, 9-control study examining expression of RAB32 in 10 μm sections, containing an acute lesion of an MS patient (referring to tissue phenotype (**g**–**i**)) and white matter from control subjects (**j**–**l**). **g** Low power image (×100 mag) of clumps of macrophages ingesting myelin stained with oil red-O within the active border of an acute lesion (fresh frozen tissue) surrounded by gray matter and normal-appearing white matter (NAWM) demarked by *dotted line*. **h** RAB32 immunostained with DAB nickel chloride localized within the cell bodies of microglial cells at low magnification (×100 mag) and **i** at higher magnification (×400). **j** Weak RAB32 expression in the blood vessel and NAWM of a control subject. **k** Intermediate RAB32 staining in glial cells present in the NAWM of a separate control subject brain section (×100 mag) and **l** at ×400 magnification. Note the intensity of staining of RAB32 in microglial/macrophage cells in acute lesions of MS patient compared to control subjects. *Scale bars* in **a**, **b**, **d**, and **e** = 50 μm and 12.5 μm in **c** and **f**

**Fig. 3:**
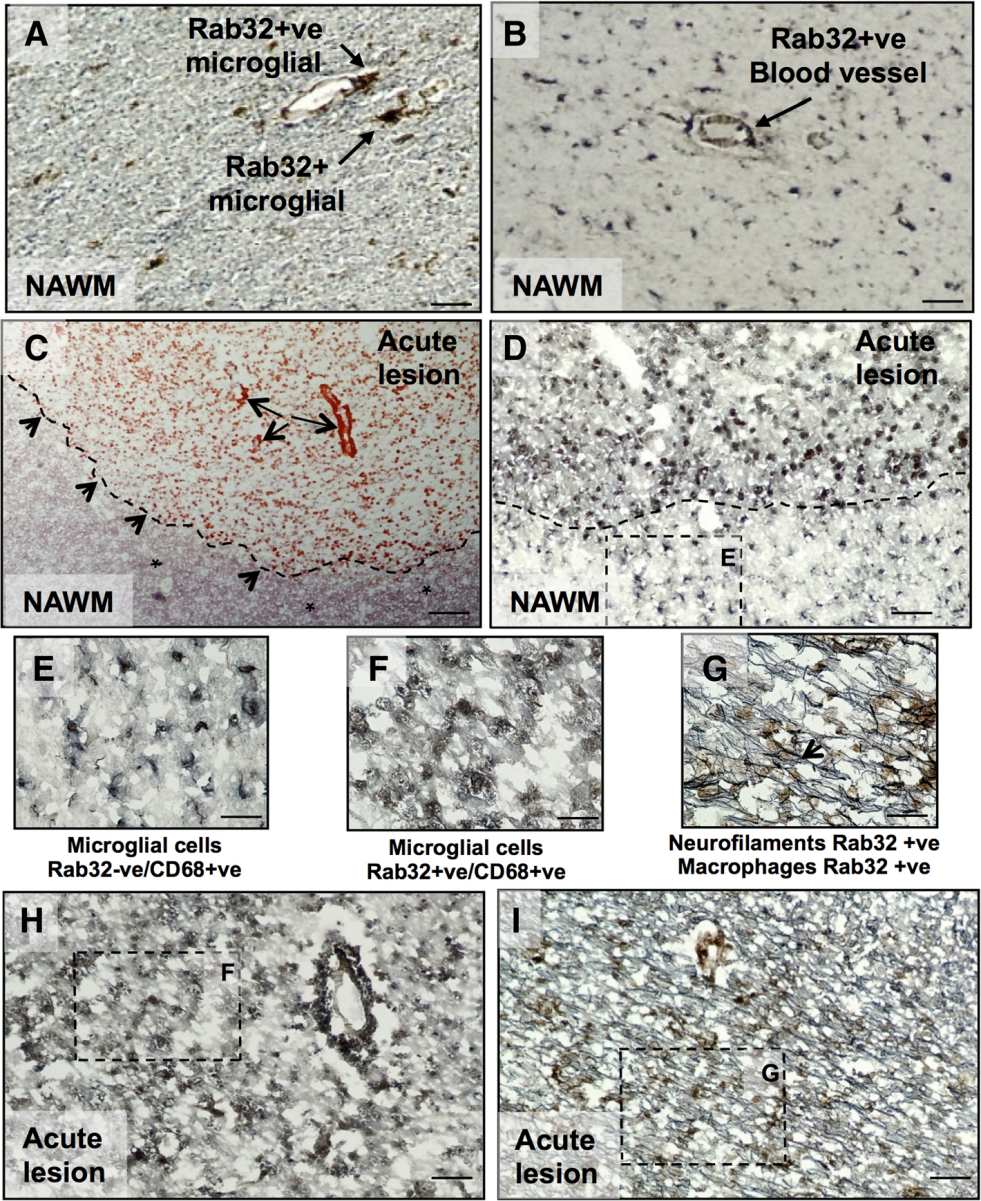
Double immunohistochemistry confirms Rab32 staining in microglia/macrophages and axons in MS. Rab32 positive staining (*brown*) was investigated in microglia/macrophages (CD68, *blue*/*gray*) and axons (neurofilament, *blue*/*gray*) with co-localization producing a black stain. **a** Staining of MS NAWM; Rab32 (*brown*) staining of CD68-positive (+ve) microglia cells. **b** Rab32 expression was also observed in microvascular cells (*arrow*). **c** Oil red-O staining of an acute MS lesion. The *dashed line* and *small arrows* depict the lesion border, and red staining shows myelin ingestion by macrophages. The *large arrows* show the blood vessels. The *asterisk* depicts surrounding NAWM. **d** Rab32-positive (+ve) microglia in the acute lesion border and Rab32-negative (−ve) microglial cells in adjacent NAWM. The *dashed lines* represent the area shown at higher power. (see high-magnification *insets E*, *F*, and *G*). **e** Rab32-negative (−ve) CD68+ve microglial cells in NAWM. **h**, **i** Staining of acute lesion tissue labeled for Rab32 (*brown*), macrophages (*blue*/*gray*, **h**), and axons (*blue*/*gray*, **i**). **f** High magnification of CD68-positive (+ve) microglial cells staining for Rab32 in acute lesions. **g** High power magnification of axons (*blue*/*gray*, co-localized with Rab32 as *black*), in close proximity to Rab32-positive (+ve) macrophages (*brown*). *Scale bars* in **a** and **b** = 25 μm; **c**, **d**, **h**, and **i** = 50 μm; and 12.5 μm in **e**, **f**, and **g**

We next examined which cell types harbor increased amounts of Rab32 in MS brain tissue and also expanded the number of patients in our study. To do so, we first stained for axonal and non-phosphorylated neurofilament that identifies cells as neurons, as well as for Rab32 and CHOP. Control brain tissue did not show significant Rab32 staining (Additional file 2). In contrast, our results shown in Fig. 2a–c demonstrate that high Rab32 expression was especially encountered at the border of active lesions of MS brains. Chronic lesions showed less expression of Rab32. In the merged images, the signals of Rab32 and neurofilament were only partially overlapping but were most pronounced in swollen axons at the active lesion border (arrows in Fig. 2a–c). Infiltrating immune cells (visible from their DAPI staining) surrounded these cells. We detected high amounts of CHOP in virtually the same set of cells that also over-expressed Rab32. Next, we evaluated to what extent microglial cells in MS brain expressed Rab32, using independent tissue samples, part of a 12-patient cohort (Figs. 2g–l and 3, Additional file 1). This showed that distinct staining for Rab32 was found within microglial cells in active lesions characterized by heavy myelin debris (stained black with DAB nickel chloride, Fig. 2g–i). Next, we discovered via double immunohistochemistry that in MS NAWM, Rab32 (brown) was localized to cells with the morphology of microglia and blood vessels, but not axons (blue/gray) (Fig. 3a, b). In contrast, in acute MS lesions (referring to tissue, Fig. 3c), we detected Rab32-positive microglia in the acute lesion border and adjacent NAWM (Fig. 3d–i). Interestingly, within the lesion area (Fig. 3h, f), we detected not only a mix of both Rab32 (brown)-positive cells and Rab32-negative macrophages (blue/gray) but also some Rab32-positive macrophages (black). Here, we also detected extensive overlap between staining for Rab32 (brown) and axons (blue/gray) (Fig. 3f–i). Together, using multiple patient tissue samples, our findings indicate that Rab32 increases dramatically in neurons and macrophages/microglia localized within active MS lesions and that high amounts of Rab32 coincide with the expression of CHOP. In contrast, chronic MS lesions show Rab32 predominantly in neurons.

### Rab32 expression is under the control of the unfolded protein response (UPR)

**Fig. 4:**
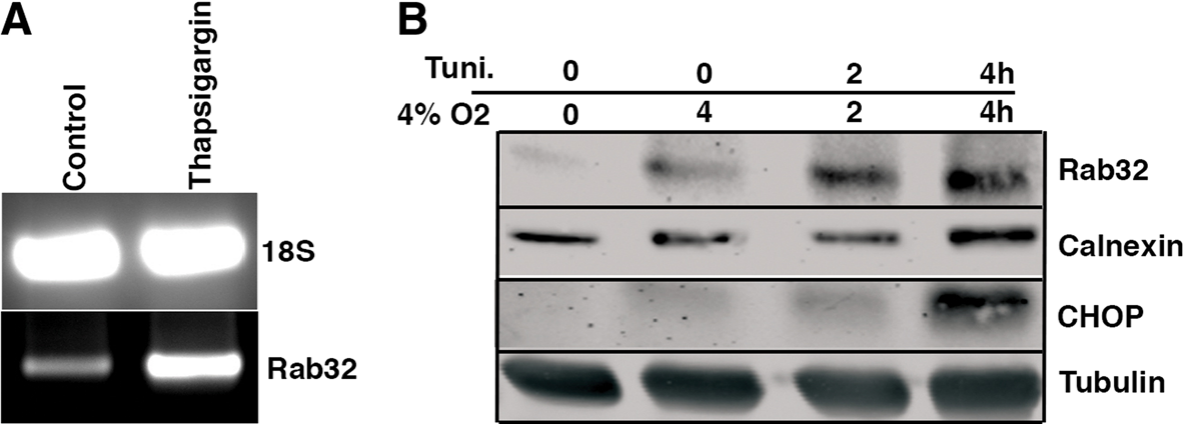
Rab32 expression under conditions of ER stress. **a** RT-PCR showing the expression of Rab32 transcripts in thapsigargin-treated SH-SY5Y cells. **b** Western blot showing the expression of Rab32, calnexin, and CHOP in tunicamycin-treated SH-SY5Y cell lines cultured in 4% O_2_ that corresponds to brain normoxia

Next, we aimed to understand what cell biological mechanism could give rise to high levels of this small GTPase. To investigate this question, we used in vitro approaches. First, we performed RT-PCR on the mRNA from SH-SY5Y cells incubated with and without 0.5 μM thapsigargin. This showed that the Rab32 mRNA increased by 2.6-fold upon ER stress (Fig. 4a). To corroborate this result at a protein level, we treated SH-SY5Y cells with tunicamycin in a 0–4-h time course under normoxic conditions or in presence of 4% oxygen. Western blotting revealed that 4% O_2_, as is typical for brain tissue, increased expression of Rab32, but tunicamycin accentuated this increase Rab32 at 2 and 4 h (Fig. 4b). In parallel, we also assessed the expression of selected ER stress-related proteins, calnexin and CHOP. Both the amounts of calnexin and CHOP were only responsive to tunicamycin treatment. Therefore, Rab32 expression appears to be tied to the induction of ER stress and to a lesser degree hypoxia, as described previously for other proteins [37].

### Rab32 interferes with neuronal mitochondrial dynamics and growth

**Fig. 5:**
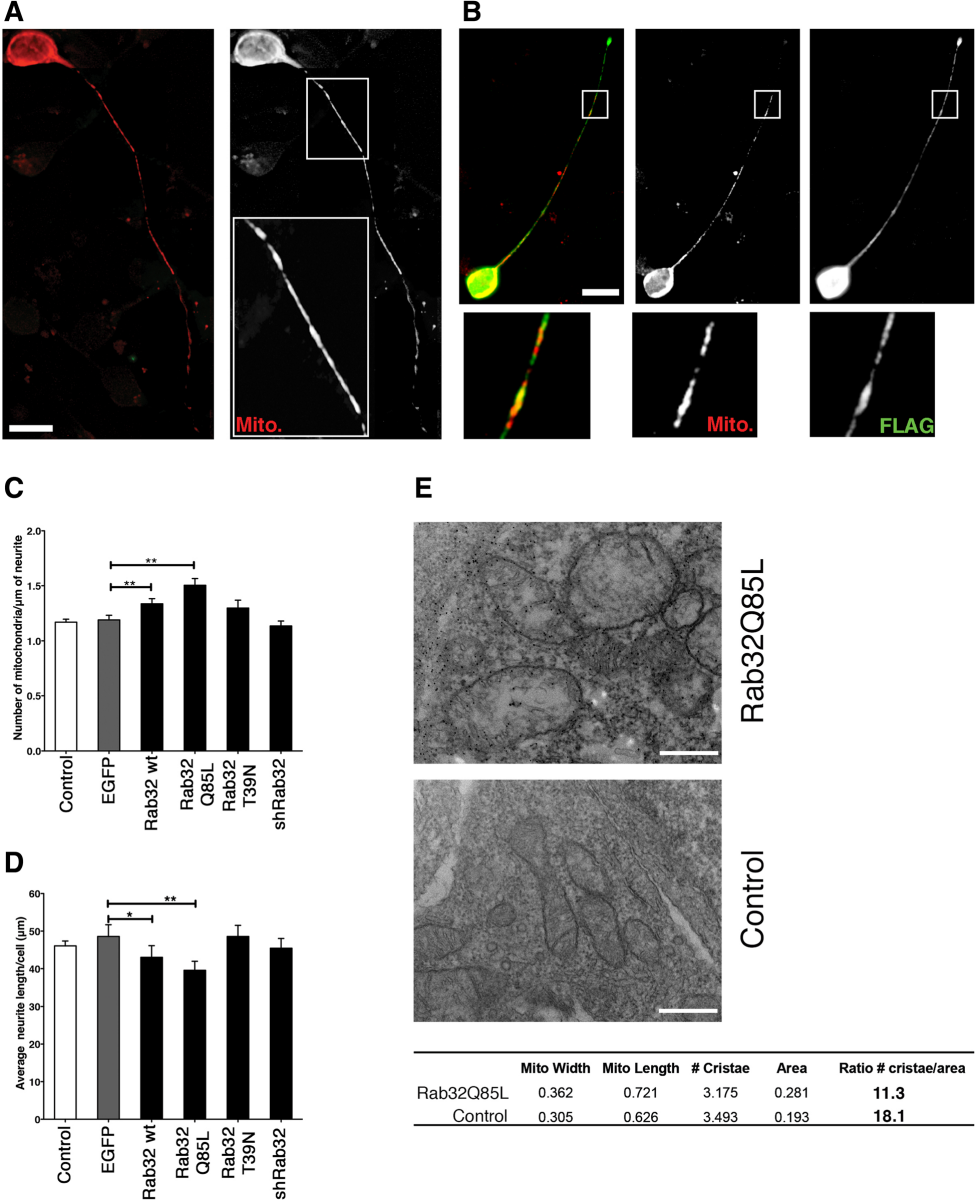
Assessment of Rab32-induced mitochondrial dynamics and neurite length in HFNs. **a**, **b** Primary human neurons were transfected with pDsRed2-Mito alone (Control: **a**) or co-transfected with pDsRed2-Mito and Rab32Q85L (**b**). Twenty-four hours post transfection, cells were processed for immunofluorescence microscopy. Mitochondria and FLAG distribution are shown as indicated. *Scale bar* 5 μm. **c** Quantification of the number of mitochondria within neurites, expressed as number of mitochondria per micrometer. **d** Quantification of the average neurite length/cell. For panels **c** and **d**, *n* = 30, *p < 0.05, and **p < 0.01, EGFP was used as an alternative control. **e** Immunoelectron microscopic determination of mitochondria phenotype. SH-SY5Y cells were transfected with FLAG-tagged Rab32Q85L, followed by immunogold detection of the FLAG signal. Mitochondria from expressing and control (non-expressing) cells were measured, and their number of cristae was determined (table below, units: μm). *Scale bar* 0.5 μm

To understand the functional readout of increased neuronal Rab32 transcription, we investigated whether Rab32 alters neuronal mitochondrial dynamics, as shown by others and us [21, 23]. Thus, we decided to express Rab32 constructs and interfering ribonucleic acid (RNAi) from bi-cistronic plasmids co-expressing nuclear EGFP. As a cellular model, we used primary human fetal neurons (HFNs) as well as SH-SY5Y neuroblastoma cells. Using the primary cells, we investigated neurite outgrowth of transfected neurons or control cells, as well as their mitochondria distribution following mitotracker-labeling. In contrast to control conditions (Fig. 5a), neurons transfected with dominant-active Rab32Q85L showed bulkier, less interconnected mitochondria units (Fig. 5b, see enlarged areas in Fig. 5a, b). Quantification revealed that transfection of neurons with Rab32WT and Rab32Q85L, but not dominant-negative Rab32T39N, indeed increased the numbers of mitochondria per length of neurite by 13 and 26%, respectively (Fig. 5c). However, this alteration of mitochondrial dynamics coincided with 12 and 22% shorter neurites in neurons expressing wild-type Rab32 and Rab32Q85L, respectively (Fig. 5d). Interestingly, knockdown of Rab32 did not have any effects for mitochondrial dynamics or neurite outgrowth, when normalized to scrambled control transfected cells (Fig. 5c, d). To investigate which effects on mitochondrial morphology resulted in the altered mitochondria density and neurite length, we transiently transfected SH-SY5Y cells with dominant-active FLAG-tagged Rab32Q85L, which showed the most significant changes. We then analyzed these cells via immunogold labeling of their FLAG signal to distinguish between transfected, over-expressing cells (top) and untransfected, control cells (bottom, Fig. 5e). This showed that Rab32Q85L promoted the formation of larger mitochondria with fewer cristae, concomitant with a 38% reduction in their cristae density per area (table, Fig. 5e). Our results imply that Rab32 alters mitochondrial dynamics in neurons and affects neurite outgrowth.

### Long-term effect of Rab32 and its mutants on neuronal survival

**Fig. 6:**
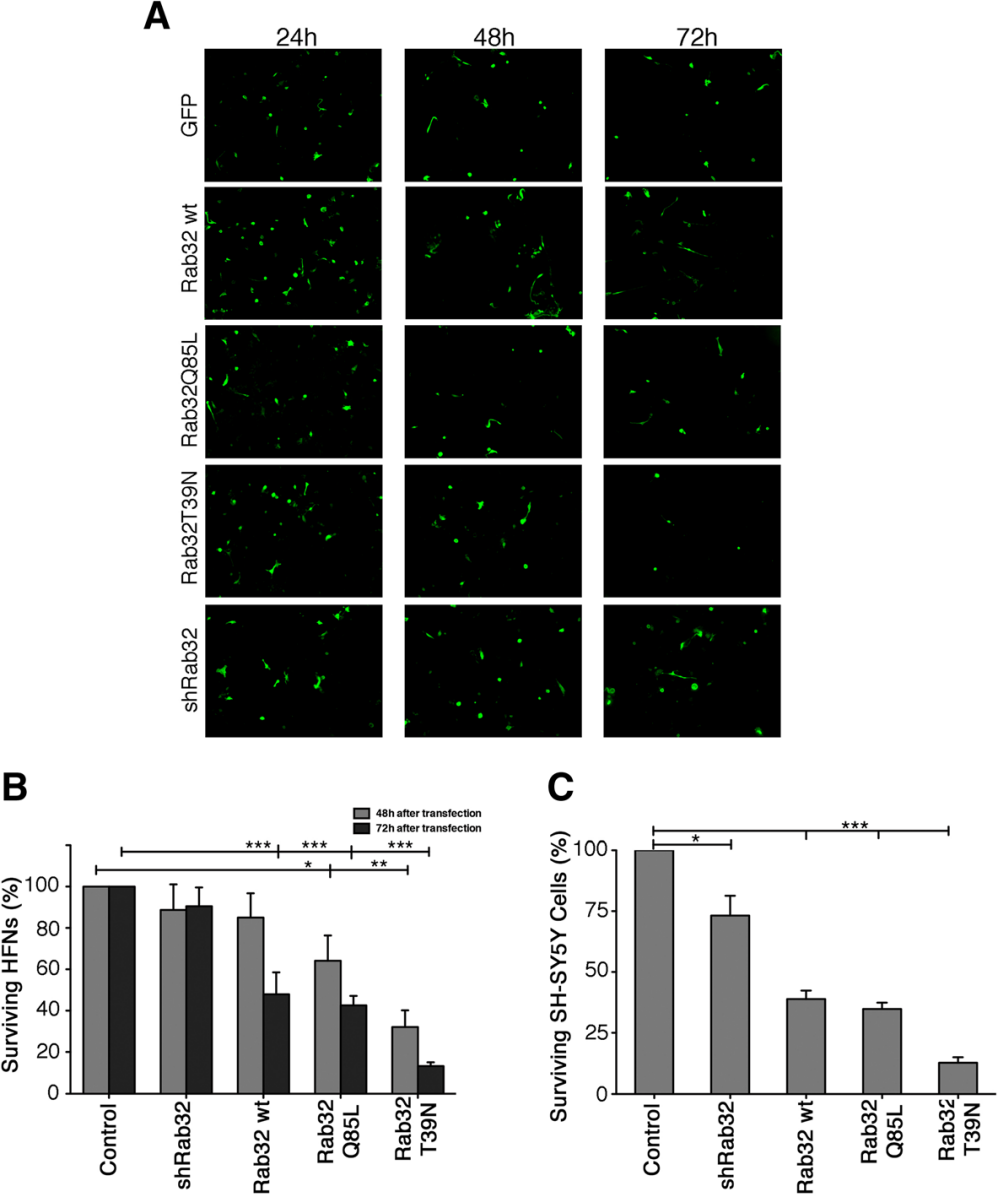
Rab32-mediated neuronal killing assay. **a** HFNs were transfected with pIRES2-EGFP, or pIRES2-EGFP expressing Flag-tagged Rab32WT, Rab32Q85L, and Rab32T39N as well as the mCherry reporter-tagged shRab32. After 24, 48, and 72 h, neurons were fixed and analyzed under a fluorescent microscopy. Note: the *red color* of mCherry was converted to *green* for the sake of consistency with the rest of the micrographs. *Scale bar* 30 μm. **b** Rab32-mediated neuronal killing was evaluated at 48 and 72 h post-transfection. **c** SH-SY5Y cells were transfected with pIRES2-EGFP, or pIRES2-EGFP expressing Flag-tagged Rab32WT, Rab32Q85L, and Rab32T39N as well as the mCherry reporter-tagged shRab32. After 24 h in culture, the cells were fixed, and the percentage of surviving neurons in comparison to the control (EGFP) was analyzed. *n* = 3; *p < 0.05; **p < 0.01; ***p < 0.001

Next, we focused on the role of Rab32 to control apoptosis onset [21] and investigated whether altered expression or activity of Rab32 would influence the survival of neurons. We assayed cell viability at 24, 48, and 72 h post-transfection and set the viability of control EGFP-expressing cells as 100%. At 24 h, we were unable to detect differences in the survival of cells with altered Rab32 expression levels or activity compared to control cells. However, the amounts of cells over-expressing any version of Rab32 started to decrease at 48 h after transfection (Fig. 6a). This trend accelerated at 72 h. In contrast, neurons expressing Rab32 RNAi as well as EGFP-only-expressing control cells did not show significant reductions in their viability (Fig. 6b). We repeated this survival assay using human SH-SY5Y neuroblastoma cells and found these cells to be even more dependent on Rab32 (Fig. 5c), regardless of whether it was active or inactive.

### Caspase inhibition and necrostatin can block Rab32-directed neuronal death

**Fig. 7:**
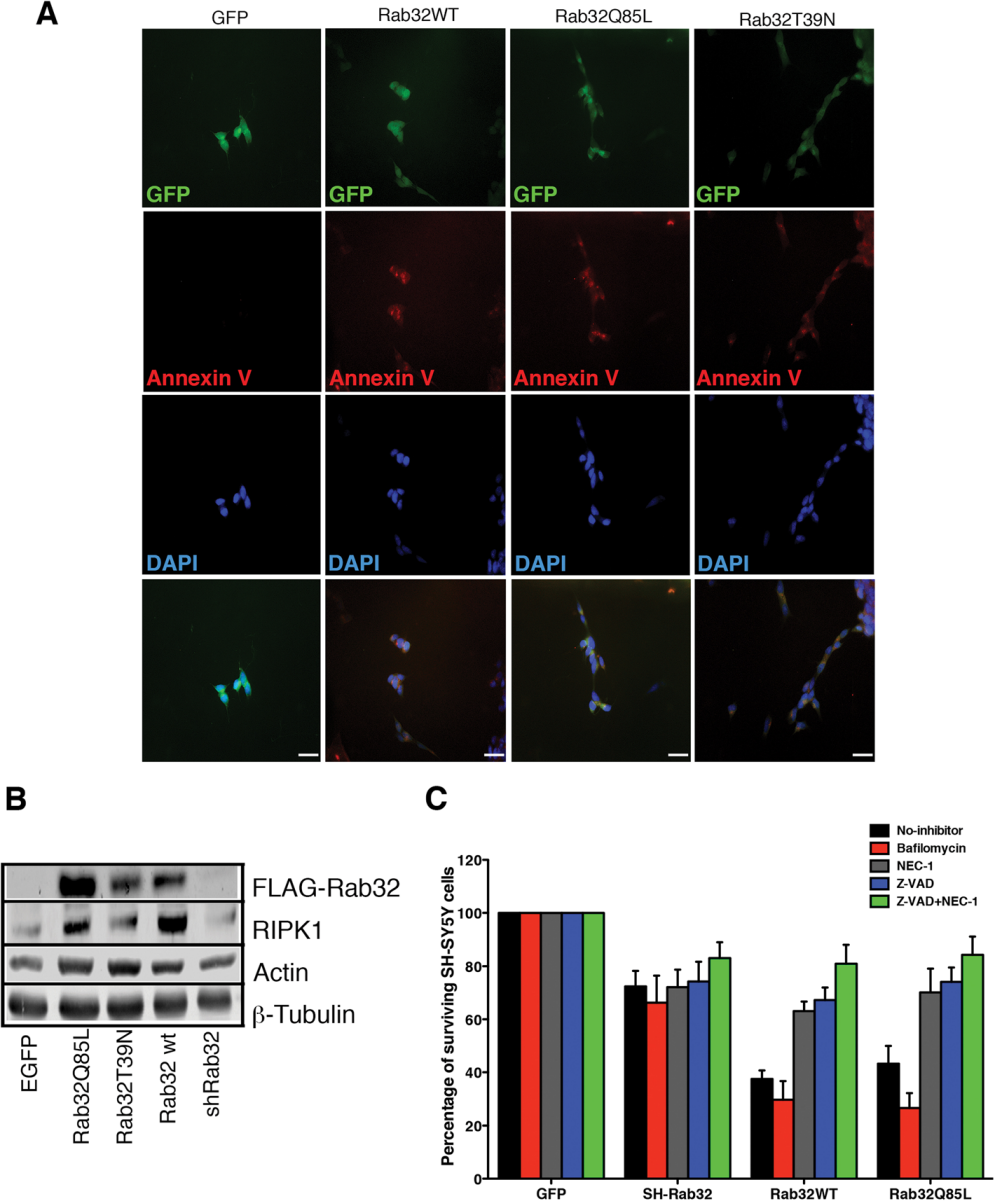
Mechanisms of Rab32-mediated neuronal killing. **a** SH-SY5Y cells were transfected with pIRES2-EGFP, or pIRES2-EGFP expressing Flag-tagged Rab32WT, Rab32Q85L, and Rab32T39N. After 24 h in culture, cells were processed for Cy5-annexin V and stained with DAPI. *Scale bars* 20 μm. **b** Western blot analysis showing the expression of Rab32, RIPK1, actin, and tubulin in SH-SY5Y cells transfected with pIRES2-EGFP, or pIRES2-EGFP expressing Flag-tagged Rab32WT, Rab32Q85L, and Rab32T39N. **c** Quantification of surviving SH-SY5Y cells transfected with pIRES2-EGFP, or pIRES2-EGFP expressing Flag-tagged Rab32WT, Rab32Q85L, and Rab32T39N either under control conditions (*black*) or treated with autophagy (bafilomycin, *red*), necroptosis (necrostatin-1, *gray*), apoptosis inhibitors (zVAD-fmk, *blue*), as well as a combination of necrostatin-1 and zVAD-fmk (*green*). After 24 h in culture, the cells were fixed, and surviving neuronal cells were quantified. In each plasmid group, each treatment was compared to the untreated control (No-inhibitor). *n* = 3

We next aimed to investigate the mechanism(s) triggered by Rab32 that led to neuronal damage and death. To do so, we transfected SH-SY5Y cells and determined whether they subsequently underwent apoptosis. Twenty-four hours after transfection, we were unable to detect annexin V on the surface of control cells (Fig. 7a, first column), but cells transfected with Rab32WT, Rab32Q85L, and Rab32T39N (identified via bi-cistronically expressed EGFP) readily showed annexin V binding (Fig. 7a, as labeled; scale bar 20 μm). In addition to apoptosis, we also investigated whether the increased expression of Rab32 might induce necroptosis. First, we investigated whether Rab32 expression and activity levels could influence the amounts of receptor-interacting protein kinase (RIPK). Thus, we lysed the SH-SY5Y cells transfected with Rab32WT, Rab32Q85L, Rab32T39N, and shRab32 as well as EGFP-expressing controls. Western blot analysis showed that increased Rab32 expression led to increased amounts of RIPK1 (Fig. 7b). No difference could be detected upon Rab32 knockdown.

To determine the relative contribution of apoptosis and necroptosis to neuronal cell death upon Rab32 over-expression, we re-examined the survival of SH-SY5Y cells transfected with EGFP, shRab32, Rab32WT, and Rab32Q85L. We then repeated our survival assay in the presence of necrostatin-1 and zVAD-fmk and, as an additional control, bafilomycin that inhibits autophagy. Quantification of the surviving cells showed that the role or Rab32 in autophagy was not responsible for our observations. In contrast, necrostatin-1 and zVAD-fmk significantly inhibited the Rab32-induced neuronal damage and death, further increased upon combination of both inhibitors (Fig. 7c). Our results therefore indicate that Rab32 induces neuronal damage and death from a combination of apoptosis and necroptosis.

## Discussion

In our study, we report that Rab32 serves as a novel marker of neurodegeneration in MS lesions, consistent with its previously detected induction in response to pro-inflammatory lipopolysaccharide (LPS) [24]. Interestingly, we found that Rab32 correlated with the inflammatory status of the tissues. In contrast to healthy tissue, which showed low levels of Rab32 as reported previously [34, 35], Rab32 was highly expressed in active lesions of both human MS patients and EAE mice; while not as high, expression of Rab32 was still elevated in chronic lesions. In terms of cell types, we have detected high amounts of Rab32 in neurons and microglial/macrophage cells.

Our investigation into a transcriptional regulation of Rab32 expression showed that this gene responds to ER stress. Since ER stress is well known to trigger inflammation and mitochondrial dysfunction, our observation that ER stress leads to Rab32 induction and subsequently alters mitochondrial dynamic as well as neuronal apoptosis induction identifies Rab32 as a protein of critical interest to MS research. Results presented in this study demonstrate that an increase of Rab32 in the inflamed brain directly promotes neuronal cell death from a combination of apoptosis and necroptosis. Interestingly, the putative role of Rab32 as an autophagy promoter [38] is not tied to this pro-death function of Rab32. While wild-type and active Rab32Q85L showed effects on mitochondrial morphology and neurite outgrowth, inactive Rab32T39N also compromised the survival of primary neurons as well as SH-SY5Y cells (Figs. 5 and 6), suggesting the mere upregulation of Rab32 is detrimental to neuronal function, potentially due to shared functions of active and inactive Rab32. Moreover, our results reinforce the role of ER stress as an upstream trigger of inflammation, which is one of the main pathology drivers in the MS context. Interestingly, the inhibition of the UPR can improve myelination of some disease models [39] and also plays a critical role in the most promising approaches to treat neurodegeneration [40].

Rab32 is induced in parallel with known mediators or regulators of the MAM, namely, Grp75, PACS-2, Mitofusin 2, and Drp1 (Fig. 1). In contrast to Rab32, however, these MAM modulatory proteins were only induced in active, but not in chronic lesions. Nevertheless, this suggests that MAM functions, including the exchange of Ca^2+^ between the two organelles and mitochondrial dynamics, actively control neuronal decay within active MS lesions but reach a new equilibrium in chronic lesions.

## Conclusions

Taken together, our results identify Rab32 as a new marker for active and chronic inflammatory lesions of the CNS in MS whose ER stress-triggered transcription leads to altered mitochondrial dynamics and neuronal cell death. Future research will have to investigate whether the inhibition of Rab32 production in the inflamed CNS could inhibit neurodegeneration in vivo.

## Additional files

Additional file 1: Information about origin of samples used for immunohistochemistry studies (Fig. 2G–L). Sample type refers to phenotype of isolated tissue as described in Methods. (JPG 810 kb)
Additional file 2: Control brain (**A**) shown CD68-positive (+ve) microglia (blue gray) but negative for Rab32 staining (brown). (**B**) Neurofilaments (blue gray) negative for Rab32 (brown). Bar = 50 μm. (JPG 1132 kb)

## Abbreviations

ALS: Amyotrophic lateral sclerosis
BiP/GRP78: Immunoglobulin-binding protein/glucose-regulated protein of 78 kDA
CHOP: CCAAT/enhancer-binding protein (C/EBP) homologous protein
CNS: Central nervous system
DAPI: 4′,6-Diamidino-2-phenylindole
Drp1: Dynamin-related protein 1
EAE: Experimental autoimmune encephalomyelitis
EGFP: Enhanced green fluorescent protein
ER: Endoplasmic reticulum
GRP94: Glucose-regulated protein of 94 kDA
GTPase: Guanosine triphosphatase
H&E: Hematoxylin and eosin
HFN: Human fetal neurons
LPS: Lipopolysaccharide
MAM: Mitochondria-associated membrane
MS: Multiple sclerosis
NAWM: Normal-appearing white matter
NC: Normal control
PACS-2: Phosphofurin acidic cluster sorting protein 2
RIPK: Receptor-interacting protein kinase
RNAi: Interfering ribonucleic acid
ROS: Reactive oxygen species
RT-PCR: Reverse transcription polymerase chain reaction
SDS: Sodium dodecyl sulphate
UK: United Kingdom
UPR: Unfolded protein response
zVAD-fmk: carbobenzoxy-valyl-alanyl-aspartyl-[O-methyl]-fluoromethylketone

## References

[1] Piaceri I, Rinnoci V, Bagnoli S, Failli Y, Sorbi S (2012). Mitochondria and Alzheimer’s disease. J Neurol Sci.

[2] Nikić I, Merkler D, Sorbara C, Brinkoetter M, Kreutzfeldt M, Bareyre FM, Brück W, Bishop D, Misgeld T, Kerschensteiner M (2011). A reversible form of axon damage in experimental autoimmune encephalomyelitis and multiple sclerosis. Nat Med.

[3] Witte ME, Mahad DJ, Lassmann H, van Horssen J (2014). Mitochondrial dysfunction contributes to neurodegeneration in multiple sclerosis. Trends Mol Med.

[4] Campbell GR, Worrall JT, Mahad DJ (2014). The central role of mitochondria in axonal degeneration in multiple sclerosis. Mult Scler.

[5] Wang X, Jiang W, Yan Y, Gong T, Han J, Tian Z, Zhou R (2014). RNA viruses promote activation of the NLRP3 inflammasome through a RIP1-RIP3-DRP1 signaling pathway. Nat Immunol.

[6] Reddy PH, Reddy TP, Manczak M, Calkins MJ, Shirendeb U, Mao P (2011). Dynamin-related protein 1 and mitochondrial fragmentation in neurodegenerative diseases. Brain Res Rev.

[7] Kageyama Y, Zhang Z, Roda R, Fukaya M, Wakabayashi J, Wakabayashi N, Kensler TW, Reddy PH, Iijima M, Sesaki H (2012). Mitochondrial division ensures the survival of postmitotic neurons by suppressing oxidative damage. J Cell Biol.

[8] Frank S, Gaume B, Bergmann-Leitner ES, Leitner WW, Robert EG, Catez F, Smith CL, Youle RJ (2001). The role of dynamin-related protein 1, a mediator of mitochondrial fission, in apoptosis. Dev Cell.

[9] Chen H, Chan DC (2009). Mitochondrial dynamics—fusion, fission, movement, and mitophagy—in neurodegenerative diseases. Hum Mol Genet.

[10] Witte ME, Geurts JJG, de Vries HE, van der Valk P, van Horssen J (2010). Mitochondrial dysfunction: a potential link between neuroinflammation and neurodegeneration?. Mitochondrion.

[11] Decuypere JP, Monaco G, Bultynck G, Missiaen L, De Smedt H, Parys JB (2011). The IP(3) receptor-mitochondria connection in apoptosis and autophagy. Biochim Biophys Acta.

[12] Patterson RL, Boehning D, Snyder SH (2004). Inositol 1,4,5-trisphosphate receptors as signal integrators. Annu Rev Biochem.

[13] Villegas R, Martinez NW, Lillo J, Pihan P, Hernandez D, Twiss JL, Court FA (2014). Calcium release from intra-axonal endoplasmic reticulum leads to axon degeneration through mitochondrial dysfunction. J Neurosci.

[14] Stirling DP, Cummins K, Wayne Chen SR, Stys P (2014). Axoplasmic reticulum Ca(2+) release causes secondary degeneration of spinal axons. Ann Neurol.

[15] Roussel BD, Kruppa AJ, Miranda E, Crowther DC, Lomas DA, Marciniak SJ (2013). Endoplasmic reticulum dysfunction in neurological disease. Lancet Neurol.

[16] Cunnea P, Mhaille AN, McQuaid S, Farrell M, McMahon J, FitzGerald U (2011). Expression profiles of endoplasmic reticulum stress-related molecules in demyelinating lesions and multiple sclerosis. Mult Scler.

[17] Bravo R, Vicencio JM, Parra V, Troncoso R, Munoz JP, Bui M, Quiroga C, Rodriguez AE, Verdejo HE, Ferreira J (2011). Increased ER-mitochondrial coupling promotes mitochondrial respiration and bioenergetics during early phases of ER stress. J Cell Sci.

[18] Csordas G, Renken C, Varnai P, Walter L, Weaver D, Buttle KF, Balla T, Mannella CA, Hajnóczky G (2006). Structural and functional features and significance of the physical linkage between ER and mitochondria. J Cell Biol.

[19] Csordas G, Varnai P, Golenar T, Roy S, Purkins G, Schneider TG, Balla T, Hajnoczky G (2010). Imaging interorganelle contacts and local calcium dynamics at the ER-mitochondrial interface. Mol Cell.

[20] Simmen T, Lynes EM, Gesson K, Thomas G (2010). Oxidative protein folding in the endoplasmic reticulum: tight links to the mitochondria-associated membrane (MAM). Biochim Biophys Acta.

[21] Bui M, Gilady SY, Fitzsimmons RE, Benson MD, Lynes EM, Gesson K, Alto NM, Strack S, Scott JD, Simmen T (2010). Rab32 modulates apoptosis onset and mitochondria-associated membrane (MAM) properties. J Biol Chem.

[22] Alto NM, Soderling J, Scott JD (2002). Rab32 is an A-kinase anchoring protein and participates in mitochondrial dynamics. J Cell Biol.

[23] Ortiz-Sandoval CG, Hughes SC, Dacks JB, Simmen T (2014). Interaction with the effector dynamin-related protein 1 (Drp1) is an ancient function of Rab32 subfamily proteins. Cell Logist.

[24] Liang Y, Lin S, Zou L, Zhou H, Zhang J, Su B, Wan Y (2012). Expression profiling of Rab GTPases reveals the involvement of Rab20 and Rab32 in acute brain inflammation in mice. Neurosci Lett.

[25] Lynes EM, Bui M, Yap MC, Benson MD, Schneider B, Ellgaard L, Berthiaume LG, Simmen T (2012). Palmitoylated TMX and calnexin target to the mitochondria-associated membrane. EMBO J.

[26] Haile Y, Simmen KC, Pasichnyk D, Touret N, Simmen T, Lu JQ, Bleackley RC, Giuliani F (2011). Granule-derived granzyme B mediates the vulnerability of human neurons to T cell-induced neurotoxicity. J Immunol.

[27] Lu JQ, Wilson B, Yong VW, Pugh J, Mehta V (2012). Immune cell infiltrates in atypical teratoid/rhabdoid tumors. Can J Neurol Sci.

[28] Lieu A, Tenorio G, Kerr BJ (2013). Protein kinase C gamma (PKCgamma) as a novel marker to assess the functional status of the corticospinal tract in experimental autoimmune encephalomyelitis (EAE). J Neuroimmunol.

[29] Holley JE, Bremer E, Kendall AC, de Bruyn M, Helfrich W, Tarr JM, Newcombe J, Gutowski NJ, Eggleton P (2014). CD20+inflammatory T-cells are present in blood and brain of multiple sclerosis patients and can be selectively targeted for apoptotic elimination. Mult Scler Relat Disord.

[30] Holley JE, Newcombe J, Whatmore JL, Gutowski NJ (2010). Increased blood vessel density and endothelial cell proliferation in multiple sclerosis cerebral white matter. Neurosci Lett.

[31] Ortiz Sandoval C, Simmen T (2012). Rab proteins of the endoplasmic reticulum: functions and interactors. Biochem Soc Trans.

[32] Murakami T, Ockinger J, Yu J, Byles V, McColl A, Hofer AM, Horng T (2012). Critical role for calcium mobilization in activation of the NLRP3 inflammasome. Proc Natl Acad Sci U S A.

[33] Zhou R, Yazdi AS, Menu P, Tschopp J (2011). A role for mitochondria in NLRP3 inflammasome activation. Nature.

[34] Bao X, Faris AE, Jang EK, Haslam RJ (2002). Molecular cloning, bacterial expression and properties of Rab31 and Rab32 New blood platelet Rab proteins. Eur J Biochem.

[35] Cohen-Solal KA, Sood R, Marin Y, Crespo-Carbone SM, Sinsimer D, Martino JJ, Robbins C, Makalowska I, Trent J, Chen S (2003). Identification and characterization of mouse Rab32 by mRNA and protein expression analysis. Biochim Biophys Acta.

[36] Deslauriers AM, Afkhami-Goli A, Paul AM, Bhat RK, Acharjee S, Ellestad KK, Noorbakhsh F, Michalak M, Power C (2011). Neuroinflammation and endoplasmic reticulum stress are coregulated by crocin to prevent demyelination and neurodegeneration. J Immunol.

[37] McMahon JM, McQuaid S, Reynolds R, FitzGerald UF (2012). Increased expression of ER stress- and hypoxia-associated molecules in grey matter lesions in multiple sclerosis. Mult Scler.

[38] Hirota Y, Tanaka Y (2009). A small GTPase, human Rab32, is required for the formation of autophagic vacuoles under basal conditions. Cell Mol Life Sci.

[39] D'Antonio M, Musner N, Scapin C, Ungaro D, Del Carro U, Ron D, Feltri ML, Wrabetz L (2013). Resetting translational homeostasis restores myelination in Charcot-Marie-Tooth disease type 1B mice. J Exp Med.

[40] Halliday M, Mallucci GR (2015). Review: modulating the unfolded protein response to prevent neurodegeneration and enhance memory. Neuropathol Appl Neurobiol.

