# Supplementary figures and images for: Rab32 connects ER stress to mitochondrial defects in multiple sclerosis
Source: J Neuroinflammation. 2017 Jan 23;14:19. doi: 10.1186/s12974-016-0788-z (PMC5260063; doi:10.1186/s12974-016-0788-z)

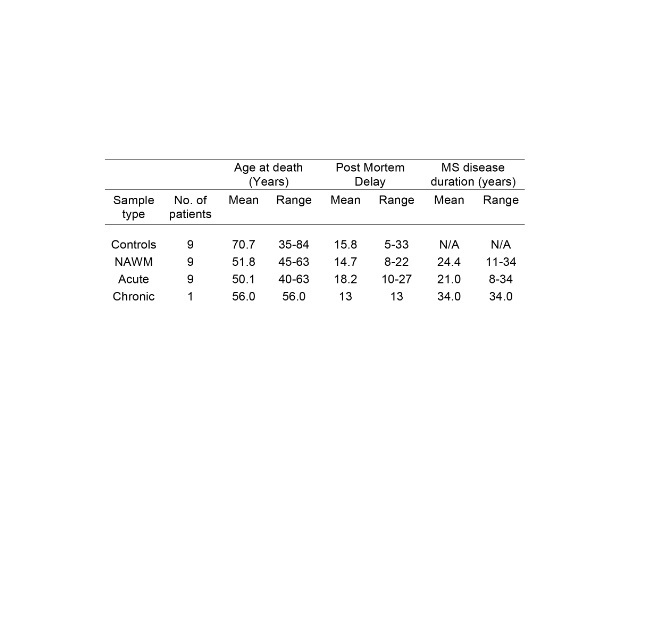

Supplement: Additional file 1: — Information about origin of samples used for immunohistochemistry studies (Fig. 2G–L). Sample type refers to phenotype of isolated tissue as described in Methods. (JPG 810 kb) [file 12974_2016_788_MOESM1_ESM.jpg]

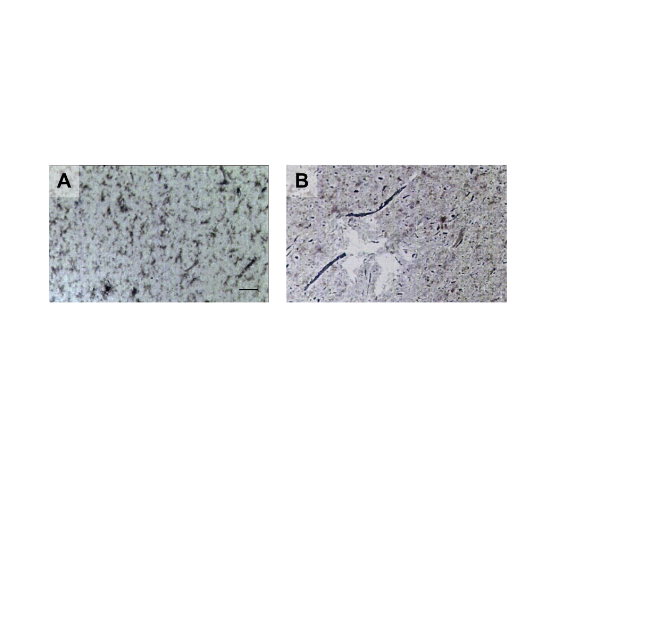

Supplement: Additional file 2: — Control brain (A) shown CD68-positive (+ve) microglia (blue gray) but negative for Rab32 staining (brown). (B) Neurofilaments (blue gray) negative for Rab32 (brown). Bar = 50 μm. (JPG 1132 kb) [file 12974_2016_788_MOESM2_ESM.jpg]
